# Metabolomic Profiles of Plant and Animal Protein and Type 2 Diabetes: Four Prospective US Cohort Studies

**DOI:** 10.21203/rs.3.rs-10525472/v1

**Published:** 2026-08-26

**Authors:** Anne-Julie Tessier, Andrea Glenn, Fenglei Wang, A. Heather Eliassen, Oana Zeleznik, Meir Stampfer, Kathryn Rexrode, Deirdre Tobias, JoAnn Manson, Clary Clish, Simin Liu, Jorge Chavarro, Frank Hu, Marta Guasch

**Affiliations:** Université de Montréal; New York University; Harvard T.H Chan School of Public Health; Brigham and Women’s Hospital; Brigham and Women’s Hospital; Harvard T.H Chan School of Public Health; Brigham and Women’s Hospital; Brigham and Women’s Hospital; Harvard T.H Chan School of Public Health; Broad Institute; Joe C. Wen School; Harvard T.H Chan School of Public Health; Harvard T.H Chan School of Public Health; University of Copenhagen

## Abstract

**Objective.:**

This study aimed to characterize plasma metabolomic signatures reflecting the metabolic pathways of plant and animal protein intake, and examine their association with the risk of type 2 diabetes (T2D).

**Methods.:**

In three cohorts, plasma metabolites were profiled from 11,742 participants from the Nurses’ Health Study (NHS; mean age 56.8 y), NHSII (mean age 44.6 y), and Health Professional’s Follow-Up Study (mean age 63.3 y) using liquid chromatography mass spectrometry. Protein intakes (% kcal) were derived from two repeated food frequency questionnaires closest to the blood draw. Elastic net regressions identified metabolomic signatures of plant and animal protein intake and their ratio. Metabolomic signatures were replicated in the Women’s Health Initiative (WHI; n = 2,092) cohort. Multivariable-adjusted Cox regression models evaluated associations between signatures and T2D.

**Results.:**

Signatures for plant protein, animal protein, and their ratio comprised 63, 50, 40 metabolites. Seventeen metabolites overlapped between the three signatures, including creatine, C34:5 phosphatidylcholine plasmalogens, C18:0 sphingomyelin, and N-acetylornithine. Higher plant protein signature scores were associated with lower T2D risk [HR per 1SD = 0.91 (0.83, 1.00); p = 0.049], while higher animal protein signature scores were associated with higher risk [HR per 1SD = 1.13 (1.03, 1.23); p = 0.008], with consistent replication.

**Conclusions.:**

Plant and animal protein intake have distinct plasma metabolomic signatures. The biological footprint of plant protein exposure is favorably associated with T2D, whereas that of animal protein is adverse. Findings from these prospective cohort studies support that increased plant protein intake may provide benefits for T2D prevention.

## INTRODUCTION

Despite long-standing dietary guidelines emphasizing the replacement of red meat with plant-derived proteins, shifting public health paradigms and popular dietary trends have reignited intense debate over the true cardiometabolic impacts of animal- versus plant-based whole foods. Yet, robust evidence from randomized trials and longitudinal cohort studies consistently supports the beneficial role of plant-based diets in improving cardiovascular health(1), lowering type 2 diabetes (T2D)(2) risk, and premature mortality risk(3, 4). Among specific protein sources, both intervention and observational studies have shown that higher red meat intake increase the risk of type 2 diabetes(5–7). Recently, a higher plant-to-animal protein ratio has been associated with a lower risk of cardiovascular disease (CVD)(8). However, because traditional nutritional epidemiology relies heavily on self-reported data, the objective biological pathways that reflect the long-term metabolic effects of habitual plant and animal protein intakes remain poorly understood.

Previous studies have suggested that unique metabolomic signatures are associated with adherence to various dietary patterns such as the Mediterranean diet(9) and plant-based diets(10). However, metabolites associated with specific protein sources have not been well-characterized(11, 12). Analyzing metabolites linked to animal and plant proteins may enhance our comprehension of biological mechanisms and explain significant distinctions between protein sources and how they are associated with cardiometabolic diseases. As emerging evidence suggests, metabolomic signatures may offer a valuable tool for understanding the metabolic consequences of differing plant and animal protein intake. This insight could advance our understanding of the biological pathways linking protein sources to cardiometabolic risk and inform dietary recommendations aimed at reducing T2D risk.

In the present study, leveraging dietary, metabolomic, and clinical data from 13,834 participants, in four U.S. adult prospective cohorts (3 discovery and 1 external replication), we aimed to identify three metabolomic signatures of dietary plant and animal protein intake, and plant-to-animal protein ratio. We also aimed to examine the prospective associations between the identified metabolomics signatures and the risk of T2D over up to 28 years of follow-up.

## METHODS

### Study population

Primary analyses were conducted in the NHS, NHSII, and HPFS ongoing prospective cohort studies. The NHS, which began in 1976, included 121,700 U.S. women registered nurses aged 30–55 years(13); the NHSII started in 1989 and involved 116,429 women registered nurses aged 25–42 years(13); and the HPFS, established in 1986, recruited 51,525 male health professionals aged 40–75 years (registered on on ClinicalTrials.gov (NCT00005182))(14). Detailed descriptions of these cohorts have been previously published(13, 14). Blood samples were obtained from 32,826 NHS participants between 1989–1990, 29,611 NHSII participants between 1996–1999, and 18,225 HPFS participants between 1993–1995. In the present analysis, we included participants who were selected for 14 prior nested case-control sub-studies on metabolomic profiling, as described elsewhere(10).

**Figure 1:**
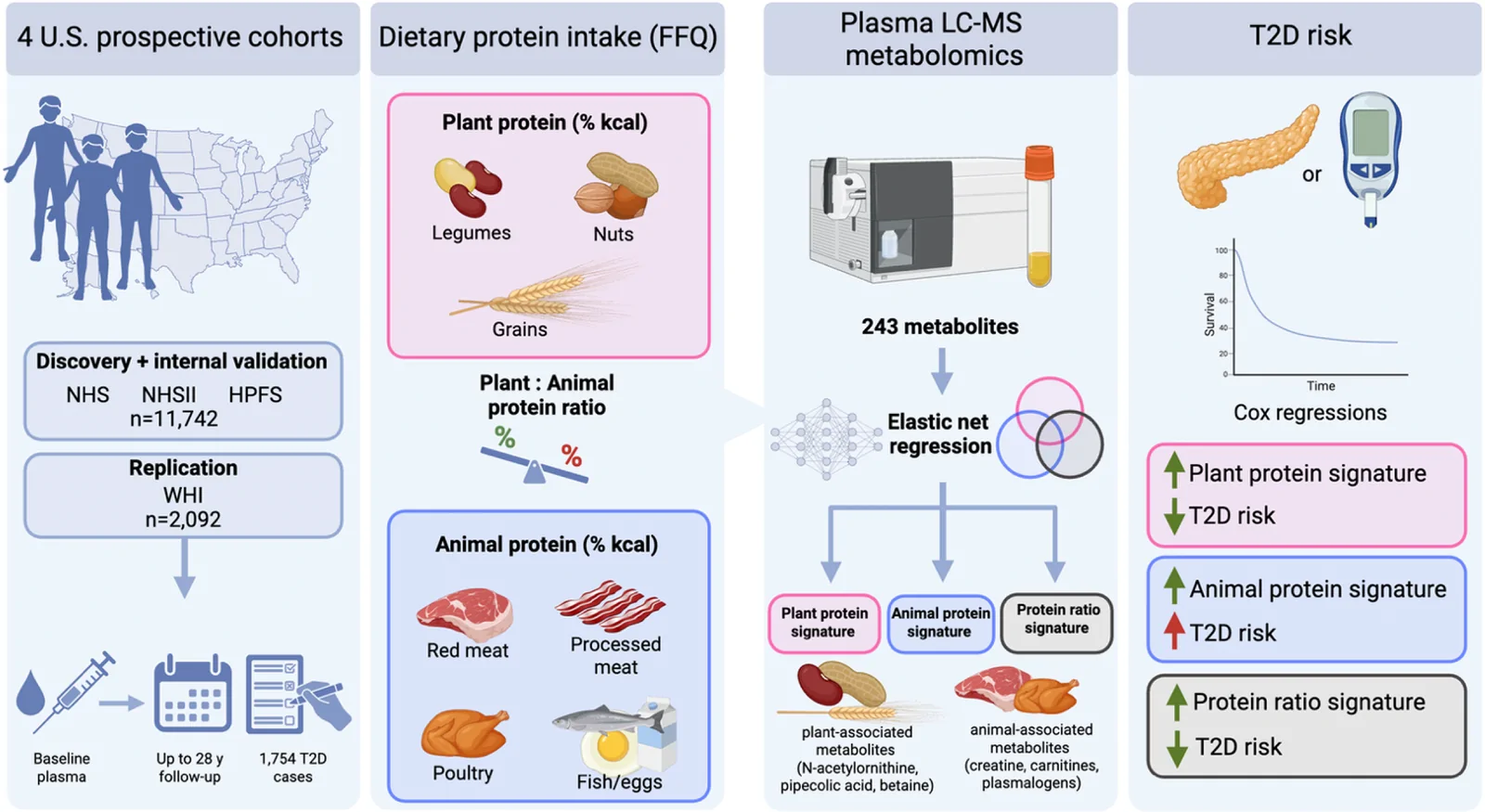
Study overview. NHS, Nurses Health Study; HPFS, Health Professionals Follow-Up Study; WHI, Women’s Health Initiative; FFQ, Food Frequency Questionnaire; LC-MS, Liquid Chromatography Mass Spectrometry; T2D, Type 2 Diabetes.

In the current study, baseline was defined as the date of blood draw for each participant. We excluded participants with a history of T2D, CVD, or cancer at baseline, those reporting implausible energy intakes (men: <800 or > 4,200 kcal/day; women: <600 or > 3,500 kcal/day), and those without available data on metabolomics profiling or diet. To allow the examination of a ratio, individuals with no animal protein consumption and those with a plant-to-animal ratio below the 1st (0.18) or above the 99th (1.41) percentiles were also excluded. A total of 11,742 participants remained in the analyses (Fig. 1). The study protocol was approved by the institutional review boards of the Brigham and Women’s Hospital and Harvard T.H. Chan School of Public Health, and those of participating registries as required.

The validation study was conducted within an external nested case-control study of coronary heart disease (CHD) as part of the Women’s Health Initiative (WHI). Initiated in 1993, the WHI included 161,808 postmenopausal women aged 50 to 79 years from across the U.S.(15, 16). Participants were enrolled either in the observational study (WHI-OS) or in the randomized controlled trials of hormone therapy (WHI-HT). At baseline, participants completed questionnaires covering socio-demographic information, diet, lifestyle, and medical history. The nested case-control study focused on 2,306 participants who had blood samples collected at enrollment(17).

For the replication analyses, individuals without metabolomic profiling and those missing dietary intake data at baseline were excluded, resulting in a final sample of 2,092 participants (Fig. 1). The study protocol received approval from the Institutional Review Board of the Fred Hutchinson Cancer Research Center in Seattle, WA, and all participants provided informed consent.

### Dietary assessment

For NHS, NHSII, and HPFS, dietary intake was assessed using a validated semi-quantitative Food Frequency Questionnaire (FFQ) administered every 4 years(18–20). Participants were asked how often, on average, they consumed specific foods and beverages over the preceding year. The mean of the two repeated measurements closest to the blood draw (one before and one after) was used for this analysis, reflecting habitual dietary intake closest to blood sample collection. For participants who did not respond to both questionnaires, the last recorded measurement was carried forward. Total protein intake (g/day) was calculated from FFQ responses by multiplying the amount and frequency of protein in each item and summing across all protein-containing foods. This same method was applied to determine the intake from animal and plant protein sources separately. The grams of protein were calculated using data from the USDA and the Harvard University Food Composition Database(21). The plant-to-animal protein ratio was calculated based on the percentage of calories from plant and animal proteins, with a larger ratio indicating higher proportion of total calories consumed from plant protein than animal protein. Intake frequencies (servings/day) for individual food groups, including processed meat, red meat, fish, poultry, eggs, dairy, legumes, nuts, refined grains, and whole grains, were also derived from the FFQ. A summary of those top food contributors to animal and plant protein in these cohorts was reported previously(8). Previous validation studies in these cohorts compared the FFQ to seven-day diet records and found a Pearson correlation coefficient of 0.61 for total protein (g/day)(20). The correlation coefficients between the FFQ and diet records in the NHS and HPFS cohorts were in the range of 0.48 to 0.74 for animal protein sources, and 0.30 to 0.45 for plant protein sources(22). A previous validation study for the WHI FFQ reported a correlation of 0.51 for total protein (g/day) compared to the mean of 8 days of dietary intake (derived from four 24-hour dietary recalls and a 4-day food record) after adjusting for energy and de-attenuating for random measurement error(23).

### Blood samples and metabolomics profiling

The plasma metabolomics profiling in all studies was performed using high-throughput liquid chromatography-mass spectrometry (LC–MS) techniques at the Broad Institute of MIT and Harvard (Cambridge, MA)(17). Polar metabolites were separated using hydrophilic interaction LC with positive ionisation mode MS detection (HILIC-pos), whereas C8 chromatography with positive ionisation mode detection (C8-pos) was used to profile lipids. Targeted raw data were processed using TraceFinder software (Thermo Fisher Scientific, Waltham, MA, USA), and non-targeted data were processed using Progenesis QI (Nonlinear Dynamics, Newcastle upon Tyne, UK). Metabolite identification adhered to the standards set by the Metabolite Standard Initiative (MSI).

A total of 404 known metabolites were identified across the Broad platforms. In our quality control steps, we excluded metabolites with WC replicates having an intraclass correlation coefficient (ICC) < 0.3 (n = 8 metabolites) or if the detection rate was < 75% (n = 153). This resulted in 243 metabolites being included in the primary analysis, primarily lipid metabolites, with known identities were measured. Metabolites were excluded if the intraclass correlation coefficient (ICC) for quality control replicates was less than 0.3 (n = 8) or if the detection rate was below 75% (n = 153). This resulted in 243 metabolites being included in the primary analysis. The metabolites primarily comprised lipids, including glycerolipids (28.8%), glycerophospholipids (12.3%), plasmalogens (10.3%), carnitines (8.2%), cholesteryl esters (CE; 4.1%), lysophospholipids (3.3%), and sphingolipids (1.6%). Other metabolite sub-classes included amines and amino acids (12.3%), nucleosides and their derivatives (4.9%), and 14% were miscellaneous metabolites. In the WHI study, from the 243 included in the discovery, 213 metabolites were available for replication analysis. For all cohorts, metabolites with skewness greater than or less than +/−2 were log-transformed. Within each laboratory analytic batch, metabolites were standardized to z-scores with a mean of 0 and a standard deviation of 1. We imputed data for participants’ missing metabolites values using the random forest imputation method(24).

### Ascertainment of incident T2D cases

In NHS, NHS II, and HPFS cohorts, participants who reported physician-diagnosed diabetes on the biennial questionnaire were sent a validated supplementary questionnaire to enquire about symptoms, diagnostic tests, and medication use, to confirm the diagnosis(25, 26). We only included confirmed type 2 diabetes cases that met at least one of the following National Diabetes Data Group criteria: (1) one or more classic symptoms (excessive thirst, polyuria, weight loss and hunger) plus fasting blood glucose ≥ 7.8 mmol/l (≥ 140 mg/dl) or random blood glucose ≥ 11.1 mmol/l (≥ 200 mg/dl); (2) no symptoms but elevated blood glucose on two separate occasions (fasting blood glucose ≥ 7.8 mmol/l [≥ 140 mg/dl] or random blood glucose ≥ 11.1 mmol/l [≥ 200 mg/dl] or 2 h blood glucose ≥ 11.1 mmol/l [≥ 200 mg/dl] after OGTT); (3) any treatment with insulin or other glucose-lowering medications for diabetes(27). The threshold for fasting blood glucose was changed to ≥ 7.0 mmol/l (≥ 126 mg/dl) as per the ADA diagnostic criteria after 1998(28).

In the WHI, for T2D, only incident cases were ascertained, defined as a self-report of physician-diagnosed diabetes treated with oral medication or insulin(29–32). Validation studies of the self-reported diabetes with use of both medical records and biomarkers indicated high accuracy and reliability(33). Time to diabetes was defined as the number of days from enrollment to the return of the questionnaire in which diabetes was first reported.

### Assessment of covariates

In the NHS, NHSII, and HPFS cohorts, participants self-reported race/ethnicity, medication, BMI, smoking, physical activity, multivitamin use, as well as the presence of diabetes, hypertension, and hypercholesterolemia during follow-up periods both before and after the blood draw. Data on total energy intake and alcohol consumption were derived from the FFQ. Overall diet quality was assessed using a modified version of the 2010 Alternative Healthy Eating Index (AHEI). This adaptation involved excluding alcohol and foods that were already accounted for in the plant-to-animal protein ratio. The modified AHEI included vegetables (excluding legumes), fruits, sugar-sweetened beverages, trans fatty acids (TFAs), polyunsaturated fatty acids (PUFAs), and sodium. For clinical disease status (hypertension, diabetes, hypercholesterolemia, cancer, MI, stroke), covariate values from the questionnaire cycle immediately preceding the blood draw were used.

For continuous covariates (BMI, physical activity), we used the mean of the two biennial questionnaire cycles surrounding the blood draw, with carry-forward imputation for missing values. In the WHI study, socio-demographic, lifestyle, and medical history information was collected concurrently with the blood draw during the initial visit. Total energy intake, alcohol, and the modified AHEI were also derived from the FFQ.

### Statistical analyses

To evaluate the metabolomic profiles associated with dietary protein intake, we applied a two-step statistical approach comprising a metabolome-wide association study of individual metabolites and the subsequent derivation of integrated multi-metabolite signatures.

#### Metabolome-wide associations.

In the NHS, NHSII, and HPFS cohorts, we examined the associations between individual metabolites (per 1-SD increment) and intakes (per 1 SD increment; %kcal) of plant protein, animal protein, and the animal:plant protein ratio using linear regressions. The models were adjusted for baseline age, fasting status, ethnicity, multivitamin use, diabetes, hypertension, antihypertensive medication use, hypercholesterolemia, lipid-lowering medication use, total energy intake, BMI, smoking status, moderate to vigorous physical activity, the modified AHEI, study cohorts, original sub-studies, and case/control status within the original sub-study. Subgroup analyses were conducted based on baseline age (< or ≥ 55 years), sex (women or men), and case-control status. The Bonferroni correction was applied for multiple comparisons (p-value/243 metabolites, p < 0.0002). To characterize the food-specific drivers of the protein source–metabolite associations, we examined associations between each metabolite and individual food group intakes (servings/day) using multivariable-adjusted linear regression. Food group intakes were standardized to z-scores (per 1-SD increment) to allow comparable effect size estimates across food groups that differ in their consumption distributions, and to facilitate direct comparison with the protein source–metabolite associations.

#### Multi-metabolite signatures.

Then, we derived metabolomics signatures of the three protein exposures using elastic net penalized linear regressions. This approach integrates both Lasso and Ridge penalties, thereby balancing variance and bias in the model. To mitigate overfitting, the data was split into training and test sets with a 70 – 30 ratio, and participants were randomly assigned to each group. First, an elastic net model (alpha = 0.5) was developed on the training set using a 10-fold cross-validation approach, selecting the lambda that minimized the cross-validation prediction error (lambda = 0.021). Then, the metabolomic signature for the test set was calculated as the weighted sum of selected metabolites, using the elastic net regression coefficients derived from the training set. This procedure was repeated 100 times, and metabolites were retained in the final signature if they were among the most frequently selected across repetitions (i.e., received a non-zero coefficient most consistently). In the training set, the signature was also obtained using a leave-one-out approach. We examined Pearson correlation coefficients were used to examine the correlations between the dietary protein metabolomic signatures and their respective self-reported dietary protein, analyzed overall, by training-test sets, and by cohorts (internal and external).

Prospective associations of the three metabolomic signatures with T2D were assessed using Cox proportional hazard ratio models in the NHS, NHSII, and HPFS cohorts. The results were validated in WHI using Cox proportional hazard models. To facilitate interpretation and comparison, both the metabolomic signatures, the self-reported protein intakes, and the ratio were standardized to z-scores (per 1SD increment). Model 1 was adjusted for fasting status and stratified by baseline age, study cohorts, original sub-studies, and case-control status within the sub-studies. Model 2 included additional adjustments for race/ethnicity, BMI, total energy intake, moderate-to-vigorous physical activity, smoking status, multivitamin use, diabetes, hypercholesterolemia, lipid-lowering medication use, hypertension, and antihypertensive medication use. Model 3 was further adjusted for the self-reported protein intake to examine the independent association of the metabolomic signature. Follow-up for each participant was calculated from baseline until the date of the last questionnaire returned, death, or the cohort-specific administrative censoring date (January 2020 for HPFS, June 2021 for NHSII, and June 2022 for NHS), whichever occurred first. Schoenfeld residuals tests indicated no violation of the proportional hazard assumption in any models. A mediation analysis was conducted to estimate the proportion of the association between self-reported protein intake and T2D incidence attributable to the metabolomic signature, for exposures where self-reported intake was significantly associated with T2D. All analyses were conducted using R version 4.1.0. The “missRanger” package was used for metabolite imputation, “glmnet” for elastic net regression, “survival” for Cox proportional-hazards models. Statistical tests were two-sided, and P values less than 0.05 were considered significant.

## RESULTS

### Participants’ characteristics

We included a total of 11,742 participants from the NHS, NHSII, and HPFS, who were predominantly middle-aged (average age 55 ± 9.2 years), women (83.5%) and white ethnicity (96.5%). Mean intake of total protein (% total calories) was reported 18.4 ± 2.7%, with 5.3 ± 1.0% from plant protein, 13.2 ± 2.9% from animal protein, and a mean plant-to-animal protein ratio was 0.43 (SD ± 0.16) (Table 1). For the WHI replication analyses, we included 2,092 participants with a mean age 67 (7). In the WHI, mean intake of total protein (% total calories) was 16.7 ± 3.1%, with 16.7 (3.1)% from plant protein, 5.0 (1.2) % from animal protein, and a mean plant-to-animal protein ratio of 0.47 (± 0.20) (Table 1).

### Metabolome-wide associations

There were 25 metabolites positively associated with plant protein intake, and 77 metabolites inversely associated (Bonferroni-adjusted P < 0.05), after adjusting for covariates. The metabolites with the strongest positive associations included proline betaine, thiamine, betaine, pipecolic acid, N-acetylornithine, along with various triacylglycerols (TAGs), mostly longer chains. In contrast, the metabolites most strongly inversely associated were mainly phosphatidylcholine (PC) and phosphatidylethanolamine (PE) plasmalogens, carnitines, and shorter saturated TAGs.

For animal protein intake, 59 metabolites were positively associated with intake and 23 metabolites were inversely associated (Bonferroni-adjusted P < 0.05), after adjusting for covariates. The metabolites with the strongest positive associations included creatine, several phosphatidylcholine (PC) and phosphatidylethanolamine (PE) plasmalogens, creatine, carnitines, branched chain amino acids, and other amino acids (tyrosine, phenylalanine, threonine, methionine), and TAGs. The metabolites most strongly inversely associated were glycine, glutamine, betaine, C18:2 LPE, pseudouridine, TAGs, and amino acid derivatives.

For the plant:animal protein ratio, 19 metabolites were positively related and 38 metabolites were inversely associated (Bonferroni-adjusted P < 0.05), after adjusting for covariates. Positively associated included C54:6 TAG and other TAGs, C36:4 DAG, thiamine, dimethylglycine, amino acids (glutamine, glycine), and derivatives (betaine, proline betaine). On the other hand, the metabolites inversely associated were several phosphatidylcholine (PC) and phosphatidylethanolamine (PE) plasmalogens, creatine, carnitines, 3-methylhistidine, some amino acids, and TAGs.

The metabolites related to protein intake were consistent across different age groups (< 55 vs. ≥55 years; Pearson r for beta coefficients = 0.88, r^2 = 0.77 for animal protein; Pearson r for beta coefficients = 0.87, r^2 = 0.76 for plant protein), sex (women vs. men; Pearson r for beta coefficients = 0.87, r^2 = 0.76 for animal protein; Pearson r for beta coefficients = 0.88, r^2 = 0.77 for plant protein), and case-control statuses (case vs. control; Pearson r for beta coefficients = 0.95, r^2 = 0.90 for animal protein; Pearson r for beta coefficients = 0.95, r^2 = 0.90 for plant protein) (**Supplementary Fig. 1**). Metabolites positively associated with dietary plant protein were inversely associated with dietary animal protein; the correlation between multi-adjusted linear regressions beta for plant protein and animal protein was −0.66 (95%CI: −0.72, −0.58; p < 2.2e-16) (**Supplementary Fig. 2**).

### Dietary protein metabolomic signatures

**Figure 2:**
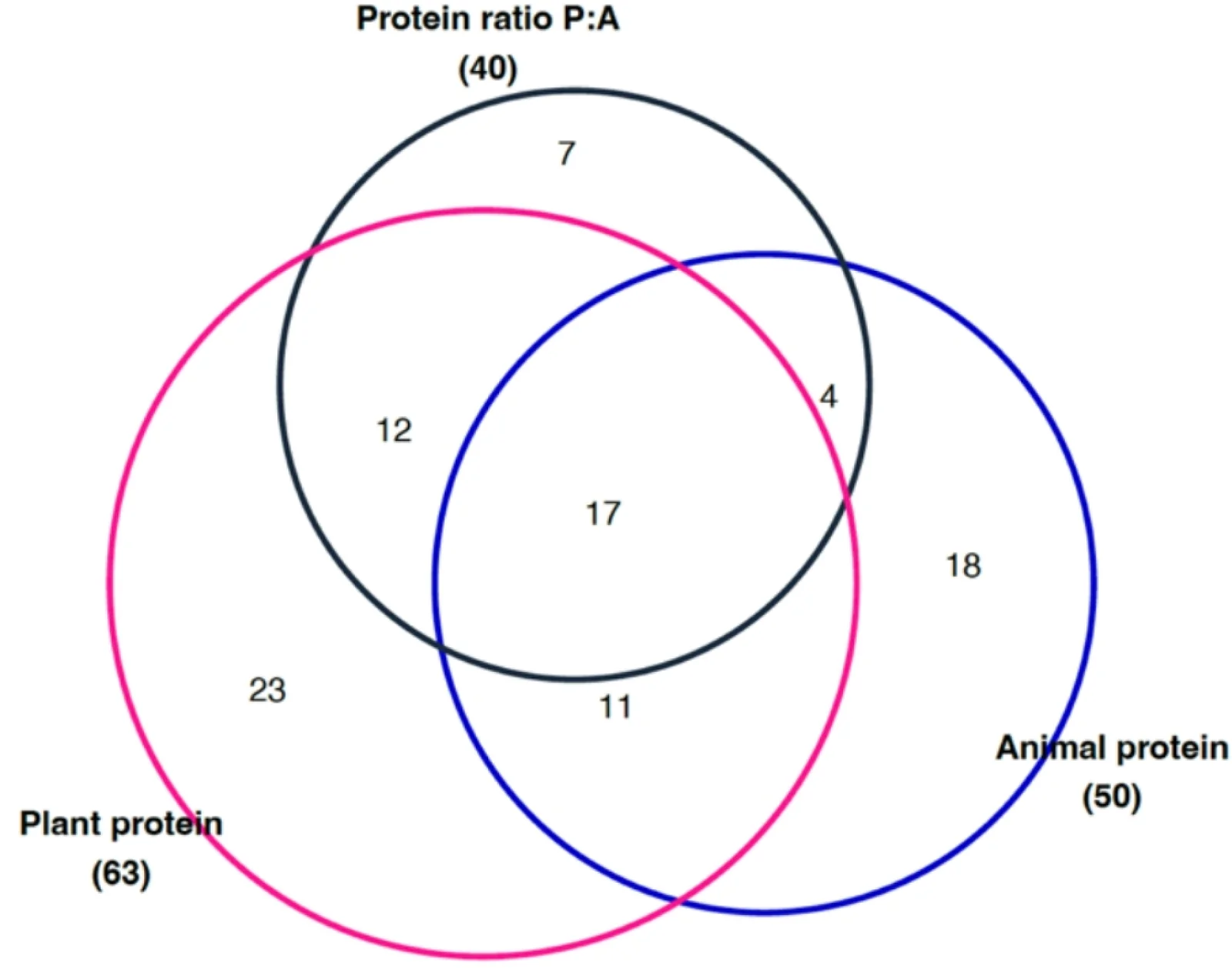
Number of metabolites retained in the protein ratio, the animal and the plant protein intake metabolomic signatures, and their overlap. P:A, Plant-to-animal protein ratio.The Euler diagram illustrates the unique and overlapping metabolites identified within the three metabolomic signatures. The area of each circle is proportional to the total number of metabolites in that respective signature. Numbers in parentheses indicate the total number of metabolites retained in each signature, while the numbers in the intersecting and non-intersecting regions represent the overlapping and unique metabolite counts, respectively.

**Figure 3:**
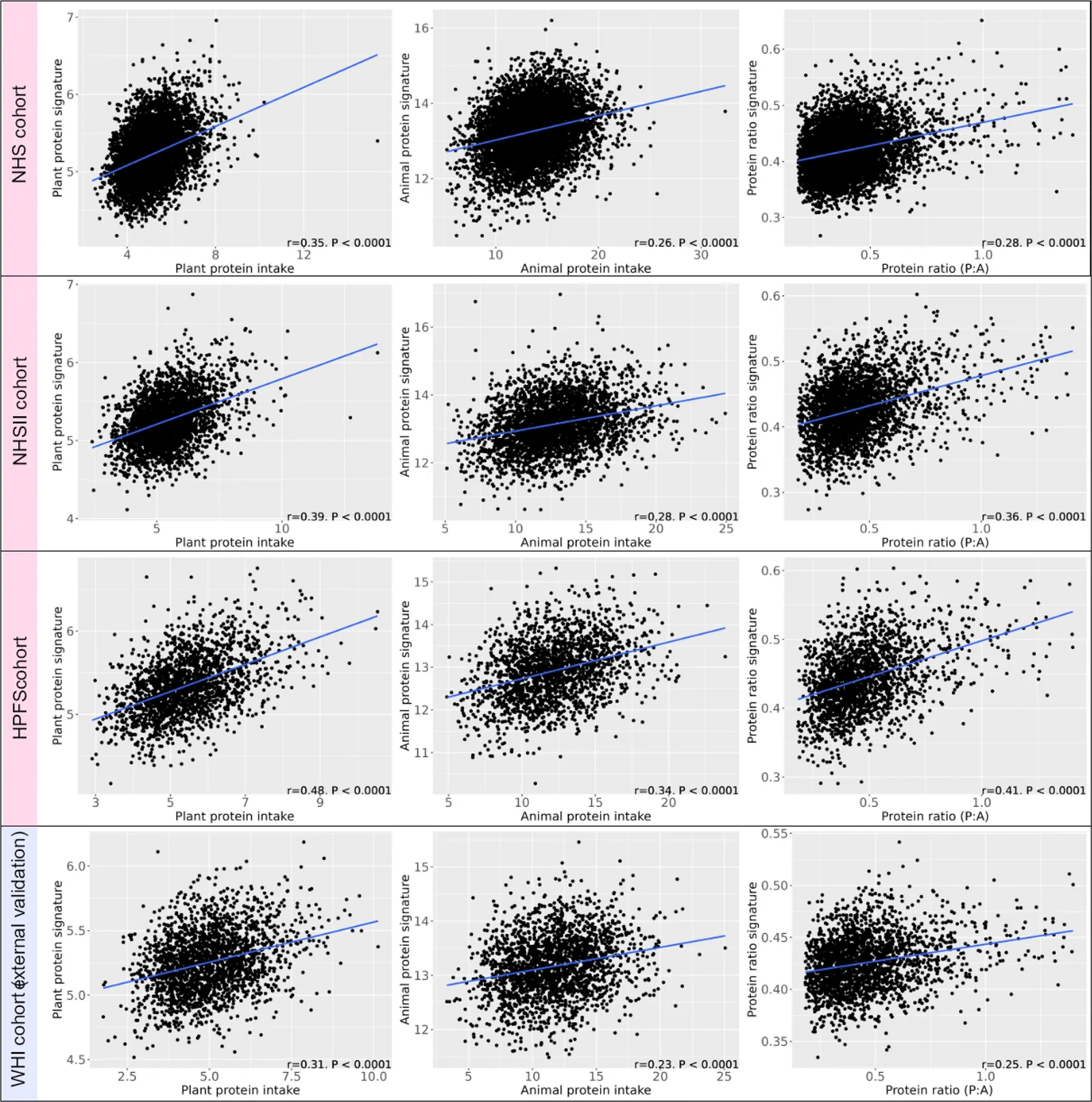
Pearson correlation of plant protein intake, animal protein intake, protein ratio, with their respective metabolomic signatures, by cohort. HPFS, Health Professionals Follow-up Study; NHS, Nurses’ Health Study; NHSII, Nurses’ Study II; P:A, Plant-to-animal protein ratio; WHI, Women’s Health Initiative. Scatter plots display individual participant data points overlaid with a linear regression line (blue line). Pearson correlation coefficients (r) and corresponding p-values (p) are shown within each panel.

**Figure 4:**
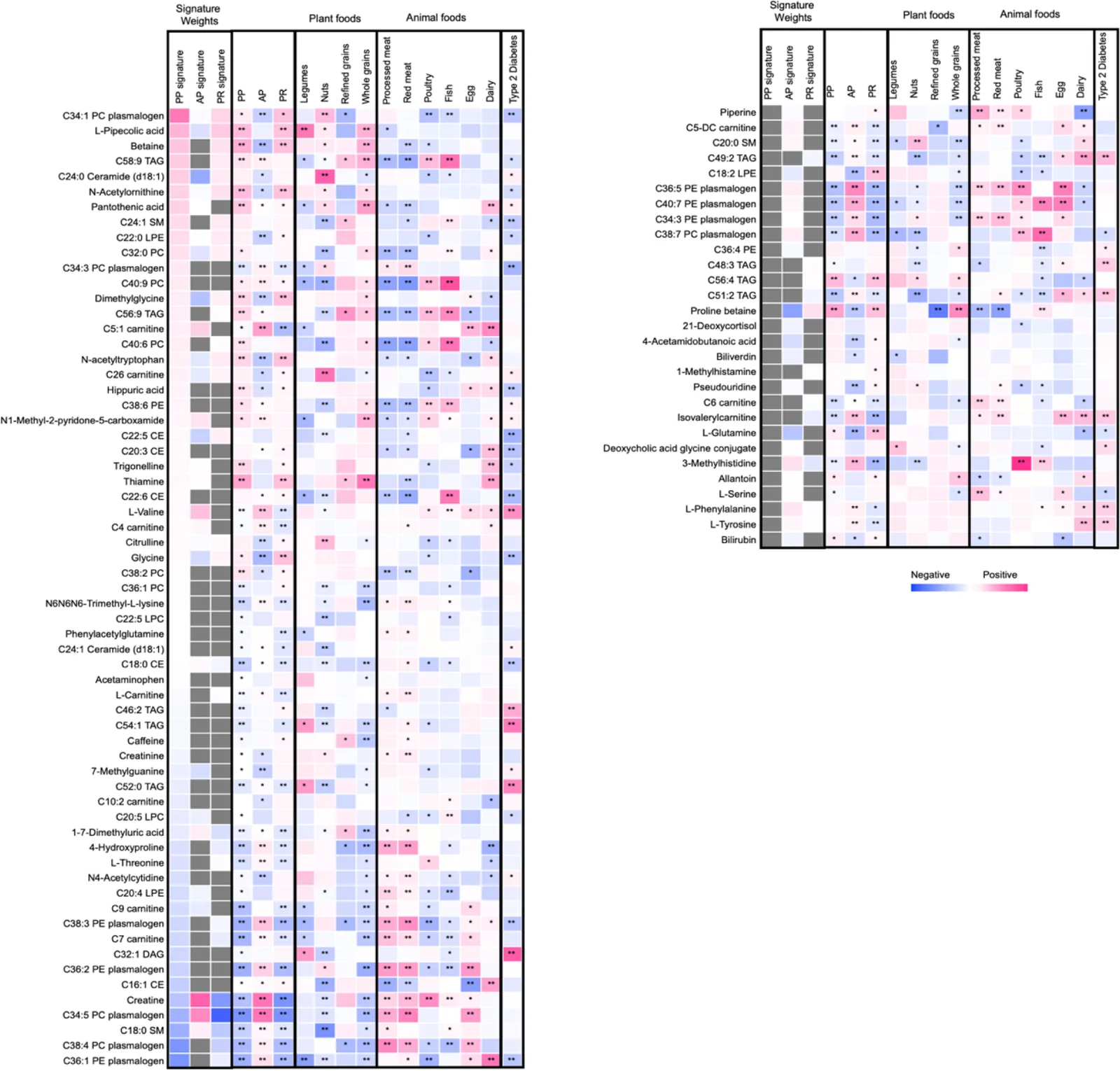
Association of individual metabolites with intake of protein-rich foods and type 2 diabetes. PR, plant to animal protein ratio; PP, plant protein intake; AP, animal protein intake. This heatmap shows the 63, 50 and 40 metabolites (total 92) retained in the PR, PP and AP signatures, their weights from elastic net regressions, β coefficients from multivariable linear regression for plant foods and animal foods (servings/d); and hazard ratios for T2D. Models are stratified for age, study cohorts, original substudies and the case/control status within the original substudy, and adjusted for fasting status, ethnicity, multivitamin use, hypertension, antihypertensive medication use, hypercholesterolemia, lipid-lowering medication use, total energy intake, BMI, alcohol intake, modified AHEI, smoking and physical activity level. *Bonferroni p < 0.05 and **p < 0.001.

In total, 63 metabolites were identified in the plant protein metabolomic signature, 50 in the animal protein metabolomic signature, and 40 in the protein ratio metabolomic signature, with 17 metabolites shared across all three dietary exposures (Fig. 2). The Pearson correlations between self-reported plant protein intake and its metabolomic signature ranged from 0.35–0.48 in the NHS/NHSII/HPFS (all p < 0.0001) (Fig. 3). The Pearson correlations ranged from 0.26–0.34 (all p < 0.0001) for animal protein intake and from 0.28–0.41 for the protein ratio (all p < 0.0001) (Fig. 3). The metabolites identified in the metabolomic signatures were predominantly associated with nuts, whole grains, and red meat (Fig. 4). Among the highest-weighted metabolites retained by the elastic net models, C34 PC plasmalogen, N-acetylornithine, and dimethylglycine were positively associated with plant protein and inversely associated with animal protein across all three signatures. Higher C34 PC plasmalogen levels were associated with higher nut intake and lower refined grains, poultry, and fish intake. Higher N-acetylornithine levels were associated with higher intake of nuts and whole grains; and higher dimethylglycine levels were associated with greater consumption of whole grains and eggs, and lower intake of dairy. Creatine and C34:5 PC plasmalogen were consistently significant contributors to all three metabolic signatures (Fig. 4) and positively associated with red and processed meat consumption, but inversely associated with nut and whole grain intake. Creatine was positively associated with all examined major sources of animal protein (processed red meat, red meat, poultry and egg), with the exception of dairy.

P:A, Plant-to-animal protein ratio.The Euler diagram illustrates the unique and overlapping metabolites identified within the three metabolomic signatures. The area of each circle is proportional to the total number of metabolites in that respective signature. Numbers in parentheses indicate the total number of metabolites retained in each signature, while the numbers in the intersecting and non-intersecting regions represent the overlapping and unique metabolite counts, respectively.

HPFS, Health Professionals Follow-up Study; NHS, Nurses’ Health Study; NHSII, Nurses’ Study II; P:A, Plant-to-animal protein ratio; WHI, Women’s Health Initiative. Scatter plots display individual participant data points overlaid with a linear regression line (blue line). Pearson correlation coefficients ( *r*) and corresponding p-values ( *P*) are shown within each panel.

PR, plant to animal protein ratio; PP, plant protein intake; AP, animal protein intake.

This heatmap shows the 63, 50 and 40 metabolites (total 92) retained in the PR, PP and AP signatures, their weights from elastic net regressions, β coefficients from multivariable linear regression for plant foods and animal foods (servings/d); and hazard ratios for T2D. Models are stratified for age, study cohorts, original substudies and the case/control status within the original substudy, and adjusted for fasting status, ethnicity, multivitamin use, hypertension, antihypertensive medication use, hypercholesterolemia, lipid-lowering medication use, total energy intake, BMI, alcohol intake, modified AHEI, smoking and physical activity level.

*Bonferroni p < 0.05 and **p < 0.001.

### Prospective associations with T2D

In NHS/NHSII/HPFS, there were 1754 incident T2D cases during a median follow-up of 20.5 years (range = 0.08–28.4). Tables 2 shows the prospective associations between the 3 metabolite signatures and T2D in NHS, NHSII, HPFS, and WHI. In the age-adjusted model, a higher baseline plant protein metabolomic signature score was associated with a 17% lower risk of T2D (HR per 1 SD; 95%CI: 0.83; 0.77, 0.91) (Table 2). A higher animal protein metabolomic signature score was associated with a 25% greater risk of T2D (1.25; 1.15, 1.36) in age-adjusted models and 13% greater risk in the fully adjusted model (1.13; 1.03, 1.23). A higher protein ratio (plant:animal) metabolomic signature score was associated with a 17% lower risk of T2D (0.83; 0.76, 0.90) in the age-adjusted model, and a 9% lower risk (0.91; 0.84, 1.00) in the fully adjusted model.

While self-reported plant protein intake and the protein ratio were not significantly associated with T2D risk in this subcohort (0.96; 0.87, 1.06 and 0.91; 0.82, 1.01), a higher self-reported intake in animal protein was associated with a 10% higher T2D risk (1.10; 1.01, 1.21). Moreover, adjustment for the animal protein metabolomic signature substantially attenuated this association. Mediation analysis further showed that the animal protein metabolomic signature explained 26.2% of the observed association between self-reported animal protein intake and T2D risk.

### External replication

In the WHI, the Pearson correlations between self-reported intake and the associated metabolomic signature for plant, animal and protein ratio signatures were 0.31, 0.23 and 0.25 respectively (Fig. 2). There were 426 incident T2D cases during follow-up. A higher plant protein metabolomic signature score was associated with a 16% lower risk of T2D (0.84; 0.74, 0.95) in the fully adjusted model (Table 2). A higher animal metabolomic signature score was associated with 28% higher risk of T2D in the replication cohort (1.28; 1.13, 1.44) (Table 2). A higher plant-animal protein ratio was also associated with a 17% lower risk of T2D (0.83; 0.74, 0.94) (Table 2).

## DISCUSSION

Our analysis of 13,942 women and men from four U.S. prospective cohorts identified three novel metabolomic signatures that offer insights into the biological processes associated with long-term plant and animal protein intake. These signatures primarily involved metabolites from the carboxylic acids and derivatives, and lipid groups. We found that a higher plant protein metabolomic signature and a higher plant-to-animal protein ratio signature were each associated with a 9% lower risk of type T2D, whereas a higher animal protein signature was linked to a 13% higher risk of T2D. In the replication cohort, a higher plant protein metabolomic signature score was associated with a 16% lower risk of T2D. Conversely, a higher animal protein metabolomic signature score was associated with a 27% higher risk of T2D. A higher plant-to-animal protein ratio was also associated with a 17% lower risk of T2D. The identified metabolic signatures could complement traditional dietary assessment tools by capturing downstream biological responses to habitual protein intake that are not reflected in intake measures alone, thereby enhancing the tailoring of dietary interventions to reduce T2D risk, as observed across these four cohorts.

Several observational studies and dietary intervention trials have examined associations or changes in circulating metabolites with omnivore versus vegetarian dietary patterns(34). Synthesizing the evidence from 16 observational studies and 8 clinical trials that used MS-based or NMR to measure metabolites, Lépine et al. reported several amino acids (including leucine, valine, glycine) and amino acid derivatives (including creatine, carnitine, proline betaine) to be associated with plant and animal protein-rich diets(34). Distinguishing between plant and animal protein sources is critical for understanding their divergent health effects, which are shaped not only by differences in amino acid composition but also by their broader food matrices. Animal proteins generally contain higher levels of branched-chain and sulfur-containing amino acids, whereas plant proteins provide distinct amino acid profiles and are typically consumed alongside beneficial nutrients such as dietary fiber, polyphenols, and unsaturated fats. In contrast, animal protein intake often co-occurs with saturated fats, heme iron, and carnitine. Metabolomic profiling is uniquely suited to capturing these holistic dietary patterns, reflecting not only protein intake itself but also the integrated metabolic signatures of accompanying compounds, including microbial metabolites and lipid fractions. However, only two studies to date have identified metabolomic profiles associated with the amount and source of protein intake(12, 35), and just one has examined associations with a plant-to-animal protein ratio(12).

In 1,833 participants at high risk of CVD (778 men and 1,055 women) in the PREDIMED trial, Hernandez-Alonso et al(12) identified 48 plasma metabolites associated with plant protein, 39 metabolites associated with animal protein, and 52 metabolites related to the plant-to-animal protein ratio using the same metabolomics platform as our study. The top metabolites associated with plant protein included C14:0 SM, C54:1 TAG, C38:4 PC plasmalogen (negative coefficients) and uridine, C34:3 PC, C22:4 CE (positive coefficients). For animal protein, the most strongly associated metabolites were choline, C18:3 CE, C46:0 TAG (negative coefficients) and creatine, C14:0 SM, GABA, sorbitol (positive coefficients). Consistent with the animal protein metabolites, the protein ratio showed C14:0 SM, GABA, C38:4 PC plasmalogen, C22:6 LPE (negative coefficients) and C34:3 PC, C22:5 CE, C20:4 carnitine, choline (positive coefficients) to be most strongly associated, likely because most protein came from animal sources. In the Atherosclerosis Risk in Communities (ARIC) study (n = 3,914 middle-aged adults, 60% women, 61% Black), Bernard et al.(35) identified 11 metabolites related to plant protein intake and 28 metabolites related to animal protein (n = 360 serum metabolites measured by gas-chromatography (GC)-MS and LC-MS). Interestingly, there was no overlap in metabolites between protein sources in ARIC. After Bonferroni correction, metabolites involved in the amino-acid, xenobiotics, and lipid super-pathways, including tryptophan betaine, 4-vinylphenol sulfate, N(delta)-acetylornithine, catechol sulfate, pipecolic acid, hippuric acid (positive coefficients) and C18:0 SM (negative coefficient), were significantly associated with plant protein intake. Metabolites of the amino acid, lipid, cofactors and vitamins, carbohydrate, and xenobiotic super-pathways, including carnitines, 2-aminobutyrate, urea, DHA, creatine, 3-methylhistidine (positive coefficients), and pyroglutamine, 1,5-anhydroglucitol, 3-methoxytyrosine (negative coefficients) were most strongly associated with animal protein intake.

In the current study, while the metabolites measured do not completely overlap with those in previous studies, several common metabolites were identified. For example, plant protein in our study was strongly associated with N-acetylornithine and pipecolic acid, both of which were also related to plant protein consumption in the PREDIMED and ARIC cohorts(12, 35). These metabolites likely reflect plant-protein consumption. N-acetylornithine is often associated with legume consumption or polyphenol-rich foods like fruits and vegetables, and pipecolic acid has been associated with dry bean intake(36, 37). These two metabolites also increased with consumption of a cholesterol-lowering plant-based diet in two controlled feeding trials that included legumes as the main protein source(38). Creatine was positively associated with animal protein intake in our study, consistent with findings from PREDIMED(12), ARIC(35), and the MASALA(39) cohorts. Valine, an essential amino acid abundant in animal protein(34), was also associated with animal protein intake in our study. Conversely, proline betaine, a marker of citrus fruit intake(36), was negatively associated with animal protein intake, likely reflecting broader dietary patterns.

Participants in our study with a higher plant protein signature and a higher protein ratio signature (indicating higher plant protein intake) had a lower risk of T2D, and a higher animal protein signature was associated with an increased risk of T2D. These results are in line with previous research, including in the same NHS/HPFS cohorts, where plasma metabolite profiles related to plant-based diets were also associated with a lower risk of T2D(10). These signatures included several overlapping metabolites, as ours related to plant and animal protein consumption, such as positive coefficients for N-acetylornithine, pipecolic acid, hippurate, and glutamine, and negative coefficients for C38:4 PC plasmalogen and creatine, among several other metabolites. Furthermore, the associations of self-reported plant and animal protein intake and T2D risk have been well studied, generally showing a protective association with plant protein sources and harmful associations with animal protein sources, particularly red and processed meats(40). Previous research has shown associations between dietary patterns that recommend plant protein over most animal protein sources, such as the Mediterranean diet, and the metabolomic signature was strongly associated with CVD risk(9). Importantly, while individual protein sources and broader dietary patterns have been evaluated, to our knowledge, no prior studies have characterized a metabolomic signature specifically for the animal-to-plant protein ratio.

Compared to previous studies(12, 35), we found more metabolites overlapping between plant protein and animal protein intake. Specifically, while no overlapping metabolites were identified between plant and animal protein profiles in the ARIC cohort (0 metabolites) and only nine were identified in the PREDIMED study, our analysis revealed 28 overlapping metabolites between these two protein sources (comprising 11 unique to the plant-animal intersection; Fig. 1). This may be due to several factors such as overall dietary patterns and populations studied.This may be due to several factors such as overall dietary patterns and populations studied. For example, the PREDIMED population(12) is Spanish and consumed more plant protein than the current US cohorts, and they are also at a higher risk of CVD. The ARIC cohort(35) is also more racially diverse than the current cohorts. Furthermore, direct comparison of research studies is still challenging when different platforms and analytic methods are used.

The study findings should be interpreted considering several limitations. First, the association between self-reported protein intake and metabolomic signatures may be bidirectional due to the cross-sectional design. Second, analyzing blood samples in batches based on case-control status could have introduced variability, though we adjusted for both batch effects and case-control status in all models. Third, because the study population primarily comprised White health professionals, the findings may lack generalizability to healthier general populations or other ethnic groups. Fourth, identical protein ratios may reflect substantially different absolute intakes and protein sources, which may carry distinct metabolic and health implications. Lastly, the study was limited to plasma metabolites of known identity, potentially biasing findings toward currently available metabolites and classes.

Despite these limitations, the study has several strengths. Our findings were reproducible across four cohorts, including men and women from various U.S. regions, with a wide range of protein intake. Robustness was supported by a large sample size, extended follow-up, and comprehensive assessment of T2D. Metabolomic signatures were developed using elastic net regularization models with train-test validation, and a leave-one-out approach minimized bias and overfitting, and the metabolomic signatures were externally validated for their association with protein intake. Metabolomic profiling was conducted consistently using start-of-the-art multi-platform LC-MS methods across all cohorts and harmonized QC protocols, including the external dataset. The broad metabolite coverage enhances future comparability and enriches understanding of metabolic pathways linking dietary protein sources and T2D. Furthermore, several metabolites in our signatures have previously been associated with plant and animal protein intake in other study populations.

In conclusion, our findings suggest that habitual intake of plant protein may induce metabolomic changes associated with a lower risk of developing T2D, whereas long-term intake of animal protein intake is associated with metabolomic profiles related to a higher T2D risk. We identified distinct metabolomic signatures associated with plant and animal protein intake in U.S. adults, providing insights into metabolic pathways underlying diet-related health outcomes. Our findings also highlight the strengths of integrating metabolomic profiling to complement traditional dietary exposure data and inform future research into metabolic pathways of T2D. Further studies are needed to validate these signatures, explore their broader applicability across populations with other protein sources, and assess their role in the prevention of other important health outcomes.

## Supplementary Material

Supplementary Files

This is a list of supplementary files associated with this preprint. Click to download.

• tessierglennsupplementals.docx

## Figures and Tables

**Table 1:** Participant baseline characteristics

Age, years	HPFS (N = 1,938)	NHS (N = 6,771)	NHSII (N = 3,033)	Total (n = 11,742)	WHI (n = 2,092)
	63.4 (8.3)	56.8 (6.8)	44.6 (4.5)	54.7 (9.2)	67.1 (6.8)
White	1843 (95.1)	6533 (96.5)	2951 (97.3)	11327 (96.5)	1697 (81.1)
BMI, kg/m	25.7 (3.2)	25.5 (4.5)	25.8 (5.4)	25.6 (4.6)	28.6 (6.0)
Current smokers	72 (3.7)	792 (11.7)	237 (7.8)	1101 (9.4)	240 (11.5)
Energy intake, kcal/d	2040 (565)	1780 (468)	1838 (500)	1838 (502)	1574 (580)
Alternative Healthy Eating Index	37.4 (7.0)	38.2 (7.0)	35.1 (6.8)	37.3 (7.1)	38.7 (8.7)
Moderate-vigorous physical activity, hours/week	4.1 (4.8)	2.2 (3.3)	3.0 (3.3)	2.7 (3.6)	1.4 (2.2)
Total protein intake, %kcal	17.6 (2.6)	18.7 (2.7)	18.4 (2.7)	18.4 (2.7)	16.7 (3.1)
Plant protein intake, %kcal	5.4 (1.1)	5.1 (0.9)	5.5 (1.1)	5.3 (1.0)	5.0 (1.2)
Animal protein intake, %kcal	12.2 (2.8)	13.6 (2.8)	12.9 (2.9)	13.2 (2.9)	11.7 (3.1)
Protein ratio (plant: animal)	0.48 (0.19)	0.40 (0.14)	0.45 (0.17)	0.43 (0.16)	0.47 (0.20)

Values are means (SDs) for continuous variables and counts (%) for categorical variables.

**Table 2:** Associations of the plant-protein, animal protein and ratio metabolomic signatures with T2D in NHS, NHSII, HPFS, and WHI.

	NHS, NHSII, HPFS		WHI	
	Metabolite signature score per 1SD increment		Metabolite signature score per 1SD increment	
	HR (95%CI)	P value	HR (95%CI)	P value
**Plant protein**				
Age-adjusted	0.83 (0.77, 0.91)	< 0.001	0.75 (0.67, 0.82)	< 0.001
MV	0.91 (0.83, 1.00)	0.049	0.83 (0.73, 0.94)	0.003
MV+mutual adjustment	0.92 (0.83, 1.01)	0.073	0.84 (0.74, 0.95)	0.007
**Animal protein**				
Age-adjusted	1.25 (1.15, 1.36)	< 0.001	1.41 (1.27, 1.56)	< 0.001
MV	1.13 (1.03, 1.23)	0.008	1.28 (1.13, 1.44)	< 0.001
MV+mutual adjustment	1.11 (1.01, 1.21)	0.026	1.27 (1.12, 1.43)	< 0.001
**Plant-to-animal protein ratio**				
Age-adjusted	0.83 (0.76, 0.90)	< 0.001	0.76 (0.69, 0.84)	< 0.001
MV	0.91 (0.84, 1.00)	0.041	0.82 (0.73, 0.93)	0.001
MV+mutual adjustment	0.93 (0.85, 1.02)	0.111	0.83 (0.74, 0.94)	0.003

HRs are per 1-SD increment. Model 1 was adjusted for fasting status and stratified by baseline age, study cohorts, original substudies, and case-control status within the original substudy. Model 2 was further adjusted for race/ethnicity, multivitamin use, diabetes, hypertension, antihypertensive medication use, hypercholesterolemia, lipid-lowering medication use, socioeconomic status, total energy intake, BMI, alcohol intake, modified AHEI, smoking and physical activity level. Model 3 further included adjustment for the respective protein source intake, or ratio to examine association independence. SD, standard deviation; HR, hazard ratio; CI, confidence interval; T2D, type 2 diabetes;

## Data Availability

Because of participant confidentiality and privacy concerns, data are available only upon written request. According to standard controlled access procedures, applications to use the NHS and HPFS resources will be reviewed by our External Collaborators Committee for scientific aims, evaluation of the fit of the data for the proposed methodology and verification that the proposed use meets the guidelines of the Ethics and Governance Framework and the consent that was provided by the participants. Investigators can expect initial responses within 4 weeks of request submission. Further information, including the procedures for obtaining and accessing data from the NHS and HPSF is described at https://www.nurseshealthstudy.org/researchers (nhsaccess@channing.harvard.edu) and https://hsph.harvard.edu/research/health-professionals/resources/for-external-collaborators/. The USDA and Harvard University food composition databases are publicly available at https://www.fns.usda.gov/usda-fis/usda-foods-database and https://hsph.harvard.edu/department/nutrition/nutrition-questionnaire-service-center/#nutrient-data.
